# O.2.1-3 Exercise Referral Schemes in the UK: mapping provision and aims

**DOI:** 10.1093/eurpub/ckad133.108

**Published:** 2023-09-11

**Authors:** Ben Jane, John Downey

**Affiliations:** Plymouth Marjon University, UK; bjane@marjon.ac.uk; Plymouth Marjon University, UK; bjane@marjon.ac.uk

## Abstract

**Purpose:**

A common physical activity intervention in the UK is that of an Exercise Referral Scheme (ERS) where a health professional is able to signpost a service user towards local physical activity opportunities, often in a local sports centre (Bully et al., 2015). While there have been a number of reviews over recent decades, the provision of ERS’s across the UK is far from standardised (Oliver et al., 2021).

The purpose of this study was to outline the provision of ERS across England and to examine how the schemes are framed and communicated in terms of their content, purpose, and approach.

**Methods:**

A database was established and systematic searching online was conducted to collect the online details of exercise referral schemes from every region in the country. Each programme’s online information was then analysed using a Content Analysis approach as it offers a “systematic, objective, quantitative analysis of message characteristics” (Neuendorf, 2016, p. 1). This method has been commonly used to engage with publicly available data in a range of web-based materials as it can be an excellent way of examining patterns and trends in communication across multiple sources (Kim & Kuljis, 2010).

**Results:**

625 unique sites were identified across 168 distinct geographical footprints. Stated delivery partners included the leisure provider (91%), at sometimes one other partner (24%). 57% were listed as being 12 weeks in duration with 10% longer and 27% not stating the duration. Many of the projects did not have a stated aim (37%) although many (36%) outlined the general benefits of being physically active. 18% identified mental health as a benefit and only 10% mentioned anything about socialising or meeting other people.

**Conclusions:**

This study found that the information publicly available on ERS schemes in England is inconsistent and often lacking in detail. Implications are that schemes should consider and then publish specific aims and objectives, and more broadly consider how they communicate the details of their programmes to stakeholders and potential participants.

**Support/Funding Source:**

No funding was received to undertake this project

